# Efficacy Evaluation of Fungus *Syncephalastrum racemosum* and Nematicide Avermectin against the Root-Knot Nematode *Meloidogyne incognita* on Cucumber

**DOI:** 10.1371/journal.pone.0089717

**Published:** 2014-02-24

**Authors:** Wen-Kun Huang, Jian-Hua Sun, Jiang-Kuan Cui, Gang-Feng Wang, Ling-An Kong, Huan Peng, Shu-Long Chen, De-Liang Peng

**Affiliations:** 1 State Key Laboratory for Biology of Plant Diseases and Insect Pests, Institute of Plant Protection, Chinese Academy of Agricultural Sciences, Beijing, P. R. China; 2 Tianjin Key Laboratory of Animals and Plants Resistance, College of Life Sciences, Tianjin Normal University, Tianjin, P. R. China; 3 Institute of Plant Protection, Hebei Academy of Agricultural and Forestry, Baoding, Hebei Province, P. R. China; Centro de Investigación y de Estudios Avanzados, Mexico

## Abstract

The root-knot nematode (RKN) is one of the most damaging agricultural pests.Effective biological control is need for controlling this destructive pathogen in organic farming system. During October 2010 to 2011, the nematicidal effects of the *Syncephalastrum racemosum* fungus and the nematicide, avermectin, alone or combined were tested against the RKN (*Meloidogyne incognita*) on cucumber under pot and field condition in China. Under pot conditions, the application of *S. racemosum* alone or combined with avermectin significantly increased the plant vigor index by 31.4% and 10.9%, respectively compared to the *M. incognita*-inoculated control. However, treatment with avermectin alone did not significantly affect the plant vigor index. All treatments reduced the number of root galls and juvenile nematodes compared to the untreated control. Under greenhouse conditions, all treatments reduced the disease severity and enhanced fruit yield compared to the untreated control. Fewer nematodes infecting plant roots were observed after treatment with avermectin alone, *S. racemosum* alone or their combination compared to the *M. incognita*-inoculated control. Among all the treatments, application of avermectin or *S. racemosum* combined with avermectin was more effective than the *S. racemosum* treatment. Our results showed that application of *S. racemosum* combined with avermectin not only reduced the nematode number and plant disease severity but also enhanced plant vigor and yield. The results indicated that the combination of *S. racemosum* with avermectin could be an effective biological component in integrated management of RKN on cucumber.

## Introduction

The root-knot nematode (RKN), *Meloidogyne* spp., is one of the most damaging agricultural pests attacking a wide range of crops [1], [2], and it can cause dramatic yield losses, mainly in tropical and sub-tropical agriculture [3], [4]. In addition to RKNs infecting economically important plants, namely cucumber and tomato in China, RKN is the most common species and perhaps the most damaging pathogen in greenhouses [5]. Approximately 50% of greenhouse-grown vegetables are infected by RKN with an annual loss caused by RKN that is estimated to be more than $400 million. In intensive cropping systems, RKN has usually been managed with chemical nematicides for decades. However, the potential negative impact on the environment and humans has led to a total ban or restricted use of most chemical nematicides [6]. With increased pressure on growers to reduce nematicide usage and without effective alternatives, there is great interest in biological control as a management tool for this destructive pathogen.

Scientists throughout the world are engaged in research to develop nonchemical and eco-friendly RKN management strategies, such as sanitation, organic soil amendments, fertilization, heat-based methods, and cultivation of resistant cultivars and transgenic plants [5], [7], [8]. Many biological antagonists, such as *Pochonia chlamydosporia* [9], *Pseudomonas fluorescens*, *Paecilomyces lilacinus* [10], *Muscodor albus* [11], and *Trichoderma harzianum* [1], have been reported to be effective in controlling RKN. Application of antagonistic microorganisms or compounds produced by theses antagonists to RKN might provide an additional option for managing RKN. However, the use of exotic antagonists that are less adapted to local climates, conditions, or target species has led to limited success in RKN control and variability of results. Therefore, it is desirable to use local species and isolates in nematode management programs [12].

The metabolites produced by the filamentous fungus *Syncephalastrum racemosum* (isolated from soil samples infected by RKN in Tianjin Province, China are potential nematode biocontrol agents against RKN, soybean cyst nematodes (*Heterodera glycines*) and the pine wood nematode (*Bursaphelenchus xylophilus*) [13], [14]. These metabolites cause more than 90% mortality of *B. xylophilus* juveniles after 120 h of exposure [14]. In greenhouse experiments, *S. racemosum* reduces multiplication rates of RKN in soil and the gall index of cucumber by 94.7 and 53.5%, respectively [15]. However, the reproductive capacity of female RKNs in roots treated with *S. racemosum* is yet to be measured under field conditions. The nematicide avermectin was first discovered in the 20^th^ century, and it has been recently used in controlling plant parasitic nematodes [16], [17]. Qiao et al. [18] reported that avermectin treatment has significant efficacy in lowering population levels of RKN compared to the untreated control. Avermectin is also effective in increasing plant height and yield as well as in reducing the incidence of *M. incognita* in potato and cucumber in field experiments [17]. However, few studies have focused on the combined effects of bioagents and avermectin.

The aim of our research was to test and evaluate the efficacy of *S. racemosum* as a biological control agent in controlling RKN on cucumber and its effect on the growth of cucumber over two successive years under field conditions. We also compared the efficacies of *S. racemosum* (fungus), avermectin (biological nematicide), and their combination to expect an increase in plant production or vigor with the combined treatment.

## Materials and Methods

### Nematode Inoculum Preparation

Eggs of RKN (*Meloidogyne incognita*) were extracted from infected roots of tomato (*Solanum lycopersicum*) using 1% sodium hypochlorite (NaClO), and the eggs were gently washed with tap water to remove the NaClO [19]. The nematode was identified on the basis of the perineal pattern of mature females [20].

### Syncephalastrum Racemosum Inoculum Preparation

The *S. racemosum* Sr18 isolate was used for all experiments and was maintained on potato sucrose malt extract agar (PSMA) plate slants. Potato sucrose (PS) liquid medium was inoculated with a spore suspension to a final concentration of 10^7^ spores per liter in a shaking flask according to the procedure described by Sun et al. [14]. Fermentations were carried out in a 30 L fermenter containing PS liquid medium with aeration (1∶0.5 vvm) and stirring (100–400 rpm) at 27°C for 42 h. Culture filtrate and mycelium were separated by filtration and adjusted to pH 7.0 with NaOH.

### Pot Experiment

The experiment was conducted in plastic pots (30 cm in diameter) filled with steam-sterilized sandy loam soil amended with organic peat and sand (field soil: organic peat: sand = 1∶3∶4). All pots (three plants per pot and 10 replicates) were arranged in a completely randomized block design on a bench in the greenhouse at 25–30°C. The treatments were as follows: (1) untreated control without RKN; (2) untreated control inoculated with *M. incognita*; (3) *S. racemosum* (2 ml); (4) 1.8% avermectin emulsifiable concentrate (EC) (0.6 ml); and (5) *S. racemosum* (1 ml) +1.8% avermectin EC (0.4 ml) (Table 1). The concentrations of *S. racemosum* and avermectin in treatments 3–5 were suspended in 400 ml of tap water and applied as a soil drench before sowing. The treatment 1 and 2 pots were irrigated with only 400 ml of tap water. Cucumber (*Cucumis sativus*) seeds were surface sterilized with 1% sodium hypochlorite (5 min) and sown directly in the pots (5 seeds per pot). The seed germination rate was calculated to be 10 d. When the cucumber seedlings reached the two-leaf stage, each pot in treatments 2–5 was inoculated with approximately 2000 second-stage juveniles (J2) of *M. incognita* by pouring 10 ml of the nematode suspension into holes with a 2–4 cm depth around the bases of the plants. Forty-five days after nematode inoculation, the cucumber plants were uprooted and washed free of adhering soil. The shoot length, root length, and number of galls per root system were measured and counted, as appropriate. The vigor of the seedlings was evaluated by calculating the vigor index (VI) [21] as follows:
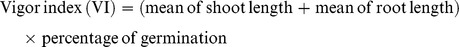



**Table 1:** Effect of biocontrol agents on seed germination and seedling vigor of cucumber plants in pots.

Treatments	Growth indices			
	Germination (%)	MRL^c^ (cm)	MSL^c^ (cm)	VI^c^
1. *M. incognita-*free control	89.3 a	19.5 a	48.3 b	60.5±5.8 b
2. *M. incognita-*inoculated control^a^	87.8 ab	15.8 b	43.3 c	51.3±2.7 c
3. *Syncephalastrum racemosum* (2 ml^b^)	86.3 ab	21.5 a	53.5 a	67.4±3.5 a
4. Avermectin (0.6 ml^b^)	81.5 b	19.0 ab	50.3 ab	56.5±5.7 b
5. *S. racemosum* (1 ml)+avermectin (0.4 ml^b^)	86.8 ab	20.8 a	49.8 ab	60.8±2.8 b
LSD 0.05	6.6	3.7	4.3	6.5

a Treatments 2–5 were inoculated with approximately 2000 second-stage juveniles at the two-leaf stage of cucumber seedlings.

b Treatments 1–2 were irrigated with 400 ml of tap water. The concentrations of *S. racemosum* and avermectin in treatments 3–5 were suspended in 400 ml of tap water and applied as a soil drench before sowing.

c MRL = mean root length, MSL = mean shoot length, VI = vigor index. The values in columns followed by the same letter(s) are not significantly different according to LSD (P = 0.05).

Meanwhile, nematodes were extracted from the soil using a Baermann pan [22]. The cucumber roots were stained with acid fuchsin [23]. Nematodes extracted from the soil or stained in the host plant roots were examined and counted under a dissecting microscope. Nematode density in the soil was expressed as the number of nematodes per 200 g of soil, and nematode density in the roots was expressed as the number of nematodes per gram of wet root.

### Field Experiment

The study was performed in a commercial greenhouse on private land located in Baodi County, Tianjin Province, China (E117°17′5′′ and N39°44′7′′; elevation of 10.6 m) in October 2010 and 2011. The field studies did not involve endangered or protected species, and no specific permissions were required for these locations/activities. Cucumber had been cultivated in this greenhouse for 10 years before our experiments. In July 2010, the disease incidence was 80–95%, and the galling index varied between 46 and 67. The soil texture was sandy loam with the following properties: pH = 7.4; electrical conductivity = 4.2 dS m^−1^; NH_4_^+^-N content = 0.7 mg kg^−1^; NO_3_^–^N content = 59.4 mg kg^−1^; total N = 1.35 g kg^−1^; extractable P = 264.5 mg kg^−1^; extractable K = 167.0 mg kg^−1^; and organic C = 8.2 mg kg^−1^.

Four experiments were designed in randomized blocks with four replicates as follows: (1) untreated control; (2) *S. racemosum* filtrate (10 ml m^−2^); (3) 1.8% avermectin EC (3 ml m^−2^); and (4) *S. racemosum* filtrate (5 ml m^−2^) +1.8% avermectin EC (2 ml m^−2^) (Table 2). Plots were 5 m long and 3 m wide (4 plant rows). Seedlings of cucumber cv. Jinglv (Tianjin Kernel Cucumber Research Institute) were produced on a field without RKN, and 15 plants were transplanted in each row. Two days before transplanting, *S. racemosum* and avermectin were dissolved in 10 L of water and used for irrigation on the planting furrow in each replicate. The untreated control was irrigated with 10 L of water only.

**Table 2:** Effect of biocontrol agents on the number of galls and second-stage juveniles in soil and roots of cucumber infected with *Meloidogyne incognita* in the pot experiment.

Treatments	Galls			Second-stage juveniles (J2) in soil			Second-stage juveniles (J2) in roots		
	Galls (x)/root system	√x+1	Decrease over control (%)	J2 (y)/200 g soil	√y+1	Decrease over control (%)	J2 (z)/g root	√z+1	Decrease over control (%)
1. Non-inoculated control									
2. Untreated inoculated control	547	23.4±0.74 a^c^		418	20.5±0.36 a		31.4	5.7±0.31 a	
*3. S. racemosum* (2 ml)	74	8.7±0.36 b	62.8	98	9.9±0.42 b	51.7	7.6	2.6±0.30 b	54.4
4. Avermectin (0.6 ml)	24	5.0±0.44 c	78.6	27	5.3±0.49 c	74.2	2.6	1.9±0.13 c	66.7
5. S. *racemosum* (1 ml)+avermectin (0.4 ml)	29	5.5±0.54 c	76.5	24	5.0±0.48 c	75.6	3.2	2.1±0.05 c	63.2
LSD 0.05		0.83			0.68			0.35	

a Treatments 2–5 were inoculated with approximately 2000 second-stage juveniles at the two-leaf stage of cucumber seedlings.

b Treatments 1–2 were irrigated with 400 ml of tap water. The concentrations of *S. racemosum* and avermectin in treatments 3–5 were suspended in 400 ml of tap water and applied as a soil drench before sowing.

c MSL = mean root length, MSL = mean shoot length, VI = vigor index. The values in columns followed by the same letter are not significantly different according to LSD (P = 0.05).

c Each transformed figure is the average of four replicates±SD. The mean in columns followed by the same letter(s) did not significantly different according to LSD (p = 0.05).

Prior to the application of biological agents, 10 soil cores (10 cm in diameter and 25 cm in depth) were collected from each plot for calculating nematode population density. Soil samples were also collected at 30, 60 and 180 d after transplanting (DAT). Second-stage juveniles of *M. incognita* were extracted from a subsample of soil (200 g) by the Baermann funnel method [24] and counted under a stereoscopic microscope. At 30, 60 and 180 DAT, 10 plants from each plot were uprooted, and the soil adhering to the roots was removed by gentle agitation in water. Root gall severity was assessed using a 0–5 rating scale according to the percentage of galled tissue (0 = 0%; 1 = 1–15%; 2 = 16–25%; 3 = 26–50%; 4 = 51–75%; and 5 = 76–100%). Roots were then carefully washed and stained with acid fuchsin [23]. Females and juveniles per gram of root were counted. Mature fruits were weighed every 4 d from all of the plants per plot until the end of the harvest.

### Statistical Analysis

Growth indices of plants, galls and Second-stage juveniles of RKN in pot experiment were used for statistical analysis. Data for total cumulative yield per month, galls and nematode number under field condition were used for statistical analysis. Analysis of variance (ANOVA) was performed for the pot experiment and the field experiment. The significant differences among the treatments were determined according to Least Significant Difference (LSD) test using SAS software version 8.0 (SAS Institute,Cary, NC) (P<0.05) [25].

## Results

### Pot Experiment

#### Effects of biological agents on seedling vigor

To determine the effect of biological agents on the vigor index of cucumber, we cultivated cucumber in the presence or absence of nematodes and biological agents. The mean root length (MRL), mean shoot length (MSL) and vigor index (VI) of the control plants inoculated with J2 of *M. incognita* were significantly reduced (p<0.05) (Table 1). The germination of the seeds was not influenced in any of the treatments. However, all treatments, except avermectin, increased the VI and MRL. All treatments increased the percentage of MSL compared to the untreated inoculated control. Compared with the non-inoculated control, only the *S. racemosum* treatment induced an increase of these growth parameters. The highest VI (67.4) and MRL (24.3 cm) occurred in the treatment of *S. racemosum.*

#### Effect of biological control agents on *M. incognita*

To determine the effect of biological agents on the gall index and nematode number, we cultivated cucumber in the presence or absence of nematodes and biological agents. Cucumber in the nematode-inoculated control showed the largest number of galls and nematodes both in soil and in roots (Table 2). All of the biocontrol treatments significantly reduced root galling compared to the nematode-inoculated control. The avermectin and *S. racemosum*+avermectin treatments yielded the smallest number of root galls. The J2 in the soil or plant roots showed a similar trend to root galls. In the soil, the highest reduction of J2 was observed in the avermectin and *S. racemosum*+avermectin treatments. The avermectin and *S. racemosum*+avermectin treatments also significantly reduced the number of J2 and females in the plant roots. However, a smaller reduction of nematodes and root galls resulted from treatment with *S. racemosum* compared to treatments with avermectin and avermectin+*S. racemosum*.

### Field Experiment

To determine the effect of biological agents on nematode number under field condition, soil samples were collected from different time after cucumber transplanting. At the beginning of the experiment, the field was uniformly infested with RKN and varied between 221 and 246 J2 per 200 g of soil. All of the treatments significantly decreased the number of nematodes in both soil and roots at 30, 60, and 180 DAT (Table 3). At 30 DAT, the avermectin and *S. racemosum*+avermectin treatments showed greater efficacy at reducing nematodes in both soil and roots compared to *S. racemosum*. At 60 DAT, the *S. racemosum* treatment showed lower efficacy at reducing nematodes in roots compared to the *S. racemosum*+avermectin treatment. No significant difference in the number of nematodes in the soil was observed among *S. racemosum*, Avermectin and *S. racemosum*+avermectin treatments. At 180 DAT, the avermectin and *S. racemosum*+avermectin treatments showed greater efficacy at reducing nematodes in roots compared to the *S. racemosum* treatment. However, the *S. racemosum* and *S. racemosum*+avermectin treatments showed lower efficacy at reducing nematodes in soil compared to the avermectin treatment.

**Table 3:** Effect of different biocontrol agents alone or in combination on the number of *Meloidogyne incognita* in soil and roots as well as symptom expression on cucumber plants in the field experiment.

Treatments	30 days after transplanting			60 days after transplanting			180 days after transplanting		
	Nematode density per 200 g soil	Nematode density per g root	Root gall index	Nematode density per 200 g soil	Nematode density per g root	Root gall index	Nematode density per 200 g soil	Nematode density per g root	Root gall index
1. Untreated inoculated control^a^	331±18.2 a	24.7±6.0 a	21.3±2.6 a	584±40.5 a	91.3±4.5 a	40.3±5.6 a	762±23.5 a	157.5±13.1 a	56.1±6.8 a
*2. S. racemosum* (10 ml m^−2^)	68±9.8 b	15.9±1.9 b	5.7±0.7 b	94±7.5 b	36.8±4.9 b	16.7±0.7 b	348±16.8 b	42.1±3.3 b	29.5±6.5 b
3. Avermectin (3 ml m^−2^)	42±7.1 c	5.3±0.9 c	4.8±1.2 b	65±10.4 b	8.6±0.8 c	11.4±1.1 c	297±17.2 c	9.3±1.5 c	22.5±1.9 c
4. *S. racemosum* (5 ml m^−2^)+avermectin (2 ml)	31±8.6 c	4.9±1.3 c	4.2±1.3 b	74±6.7 b	8.1±1.0 c	10.9±1.5 c	341±11.8 b	11.7±0.7 c	21.8±3.3 c
LSD 0.05	18.1	5.0	2.5	33.1	5.2	4.5	27.4	10.5	7.9

a The *S. racemosum* and avermectin products in treatments 2–4 were dissolved in 10 L of water and used for irrigation on the planting furrow in one replicate. The untreated control was irrigated with 10 L of water only.

The reduction in root galling followed the same trend as the nematode number in soil and roots. All of the treatments significantly decreased the gall index compared to the untreated control at all observation times. At 30 DAT, no significant difference was observed among the treatments. At 60 and 180 DAT, the lowest gall index was registered after application of the avermectin and *S. racemosum*+avermectin treatments, and this index was significantly different from the *S. racemosum* treatment (P<0.05).

### Cucumber Yield

**Figure 1:**
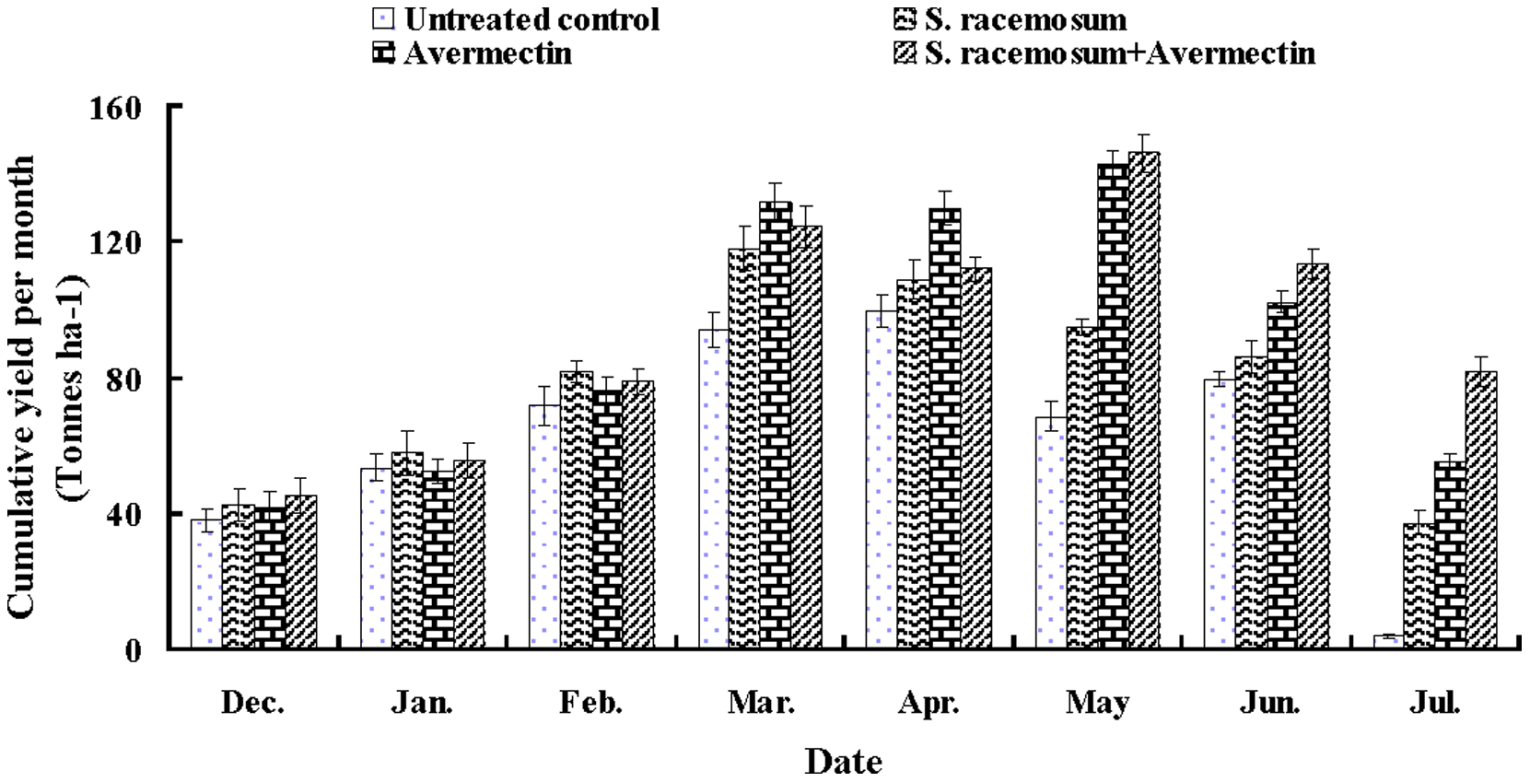
Final cumulative fruit yield per month of cucumber from December 2010 to July 2011 in the field experiment. Four experiments were designed in randomized blocks with untreated control, *S. racemosum* filtrate 10 ml m-2 (*S. racemosum*), 1.8% avermectin EC 3 ml m-2 (Avermectin) and *S. racemosum* filtrate 5 ml m-2+1.8% avermectin EC 2 ml m-2 (*S. racemosum*+Avermectin).

To determine the effect of biological agents on the yield of cucumber, cumulative cucumber yields per month were recorded from December 2010 to July 2011 (Figure 1). At the initial stage (December 2010 to March 2011), the yield did not differ significantly between the treatments and the untreated control. From midseason until the end of harvest time (April to July 2011), the yields in all treated plots were higher than in the untreated control. The highest overall yield was recorded in plots treated with *S. racemosum*+avermectin, with a mean of 756.6 t ha^−1^, followed by the avermectin treatment, with a total mean of 731.6 t ha^−1^. The lowest overall yield was recorded in the untreated control with a total mean of 508.5 t ha^−1^. The total yield after the *S. racemosum*, avermectin and *S. racemosum*+avermectin treatments was increased by 23.2, 43.9, and 48.8%, respectively. A significant yield increase during the entire season was also observed in the *S. racemosum*+avermectin and avermectin treatments compared to the *S. racemosum* treatment (p<0.05). No significant difference in the cumulative yield was observed between the treatments of *S. racemosum*+avermectin and avermectin(p>0.05).

## Discussion

Research on the use of antagonists to control plant parasitic nematodes is receiving increasing attention [26], and *S. racemosum* has been studied very little. The results from pot and field experiments in this study showed that the combination of *S. racemosum* and avermectin can cause the paralysis of RKN nematodes and reduce the application of pesticides. The decrease of final cost and the increase of plant yield may help to increase the farmers’ income. Therefore, *S. racemosum* and avermectin are promising biological agents in the integrated control of plant parasitic nematodes.

The combination of *S. racemosum* and avermectin reduced the number of galls and developmental stages in cucumber roots, which was consistent with previous field results [15], [27], [28], [29]. Sun et al. [27] reported that *S. racemosum* reduces the population density of *M. incognita* by 82.7% on tomato plants in a greenhouse. Secondary metabolites produced by *S. racemosum* were shown to cause a significant reduction of the reproduction of *M. incognita* on cucumber in a field experiment [15]. A previous *in vitro* experiment showed that *S. racemosum* metabolites inhibit hatching and reduce development of *M. incognita*, *H. glycines* and *B. xylophilus* [14], [28]. More recently, Huang et al. [29] reported that a combination of *S. racemosum*, mustard biofumigation and solarization is effective in reducing *M. incognita*, which subsequently enhances cucumber growth. However, little attention had been paid to the effects of bioagents on plant vigor and yield. Present findings have confirmed that the highest VI and MRL were obtained after application of *S. racemosum*, which suggests that *S. racemosum* may have stimulative effects on both shoot growth and plant vigor. The efficacy of *S. racemosum* on the yield also demonstrated that *S. racemosum* had a significant promoting effect on the growth of cucumber. The widely recognized mechanisms of bioagents include the production of toxins, enzymes and other metabolic products, as well as the promotion of plant growth and induction of systemic resistance of host plants to pathogens [30]. In this study, the reduction of the gall index caused by the strain may be attributed to secondary metabolites produced by the fungus in the soil and direct parasitism on nematode eggs, which may have also induced the plant defense mechanism leading to systemic resistance [1], [10]. The beneficial effects induced by *S. racemosum* combined with avermectin on the seedling vigor index and fruit weight may be attributed to the combined effect of production of growth-promoting substances and antibiotic metabolites effective against the microbial community [31].

Many fungi and their metabolites are known to possess nematicidal activity against a wide range of plant-parasitic nematodes including RKNs [3], [26], [32]. In the present studies, the antibiotic metabolites of *S. racemosum* showed good nematicidal activity against *M. incognita*. However, little is known about the nematicidal mechanisms of *S. racemosum* against RKNs despite excellent toxicologic actions. For RKNs, J2 move freely in the soil before infecting host plants, and their movement is regulated by active compounds [33], [34]. Avermectin is a streptomycete-derived macrocyclic lactone biological nematicide that blocks the transmittance of electrical activity in nerves and muscle cells by stimulating the release and binding of gamma-aminobutyric acid at nerve endings [16], which causes paralysis of the neuromuscular systems that adversely affect nematode hatching and movement in soil, subsequently reducing the extent of root invasion [32]. Secondary metabolites of *S. racemosum* may inhibit the incubation of eggs after the RKN attach to the roots of cucumber plants, resulting in reduced density of nematode populations in the soil. Therefore, the combination of *S. racemosum* and avermectin can not only cause the paralysis of nematodes in soil but can also reduce the infection of nematodes in roots. However, the exact mechanism of disease defense by this strain needs to be verified in future research.

Controlling root-knot nematodes is of major economic importance to the agricultural industry of northern China. The current study provides evidence that soil drench with the tested bio-products,avermectin and *S. racemosum* effectively reduced the number of galls and juveniles of RKN and consequently enhanced cucumber growth. The obtained results are highly encouraging, demonstrating their promising candidates as an alternative for the control of RKN in cucumber under glasshouse conditions. Further research is required to identify nematicidal components of *S. racemosum* and their mechanism of action that may be used as a part of an integrated strategy with other control methods, such as biofumigation, solarization, soil amendments, trap cropping and chemical nematicides.
